# Physiological Limits along an Elevational Gradient in a Radiation of Montane Ground Beetles

**DOI:** 10.1371/journal.pone.0151959

**Published:** 2016-04-04

**Authors:** Rachel A. Slatyer, Sean D. Schoville

**Affiliations:** 1 School of Biosciences, University of Melbourne, Parkville, Australia; 2 Bio21 Institute, University of Melbourne, Parkville, Australia; 3 Department of Entomology, University of Wisconsin-Madison, Madison, Wisconsin, United States of America; University of Cincinnati, UNITED STATES

## Abstract

A central challenge in ecology and biogeography is to determine the extent to which physiological constraints govern the geographic ranges of species along environmental gradients. This study tests the hypothesis that temperature and desiccation tolerance are associated with the elevational ranges of 12 ground beetle species (genus *Nebria*) occurring on Mt. Rainier, Washington, U.S.A. Species from higher elevations did not have greater cold tolerance limits than lower-elevation species (all species ranged from -3.5 to -4.1°C), despite a steep decline in minimum temperature with elevation. Although heat tolerance limits varied among species (from 32.0 to 37.0°C), this variation was not generally associated with the relative elevational range of a species. Temperature gradients and acute thermal tolerance do not support the hypothesis that physiological constraints drive species turnover with elevation. Measurements of intraspecific variation in thermal tolerance limits were not significant for individuals taken at different elevations on Mt. Rainier, or from other mountains in Washington and Oregon. Desiccation resistance was also not associated with a species’ elevational distribution. Our combined results contrast with previously-detected latitudinal gradients in acute physiological limits among insects and suggest that other processes such as chronic thermal stress or biotic interactions might be more important in constraining elevational distributions in this system.

## Introduction

Studies of environmental gradients are critical to developing a mechanistic understanding of how biotic and abiotic factors regulate species diversity and distributions [1]. Some of the steepest environmental transitions in nature are found along elevation transects in mountains, where abiotic factors such as temperature, humidity and solar radiation change rapidly with elevation, providing opportunities to explore how populations and species respond to macroclimatic variation [2–4]. Elevational gradients are also associated with considerable changes in community composition (elevational turnover) over small spatial scales [5]. A central question in ecology and biogeography is identifying which factors underlie species’ range limits, and thus species turnover, along elevational gradients.

In the absence of physical barriers, range limits are expected to reflect the limits of the fundamental niche, describing the resources required for a species to persist in a given environment [6, 7]. Although factors such as dispersal ability and biotic interactions can constrain species to a subset of their potential range (i.e. the realised niche [6–9]), physiological limits–as a component of the fundamental niche–are considered to be particularly relevant in mountain environments and are frequently inferred as proximate drivers of elevational range limits [10–13]. Concurrent gradients in temperature and humidity create an increasingly cold and xeric environment towards higher elevations [4]. These gradients favour lower thermal limits and greater desiccation resistance with increasing elevation [14, 15]. Insects are likely to be particularly sensitive to such changes in environmental conditions because of their small body size and the direct impacts of the external environment on their physiological processes [16, 17]. If physiological constraints determine a species’ elevational range, tolerance limits should be statistically associated with climatic characteristics at the range edge; species occupying different elevational ranges would thus exhibit differences in tolerance limits [13, 18–20].

**Fig 1:**
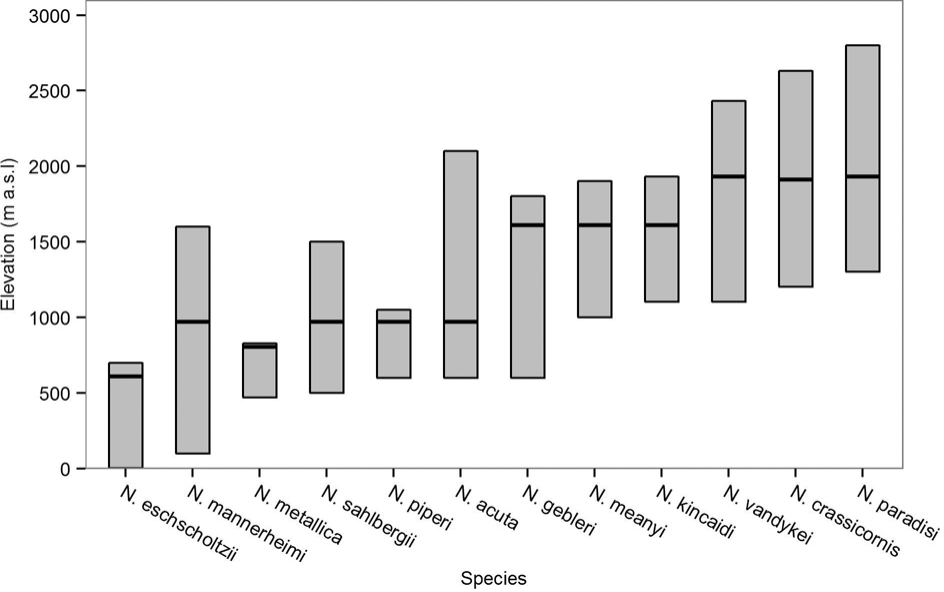
Elevational ranges and sampling elevations for study species. Approximate elevational ranges for the 12 *Nebria* species on Mt. Rainier, Washington. Range edges were derived from Kavanaugh [21] and our own transect surveys. Horizontal lines indicate the elevation of the population used in the study.

In this study, we examine whether members of the Mt. Rainier ground beetle (Coleoptera: Carabidae) assemblage, occupying different positions along a large elevational gradient, vary in their tolerance of temperature and desiccation stress. Members of the genus *Nebria* Latreille (1802) are a ubiquitous element of the Nearctic mountain fauna and 12 species occupy overlapping elevational distributions from sea level to 2750 m above sea level (a.s.l) on Mt. Rainier, Washington ([21] Fig 1). The *Nebria* assemblage also occurs on mountains to the north and south of Rainier; species occupy the same relative positions along the elevational gradient, but some species are absent at southern latitudes. This species radiation, in which cryophily is the norm, provides a compelling test of whether physiological tolerance drives species turnover along elevational gradients, and the role that microhabitat selection might play in moderating spatially varying selection. Carabids have been proposed as useful bioindicators for environmental change, with both temperature and humidity being invoked as range-limiting factors for carabids and for *Nebria* specifically [22–24]. We test the hypothesis that temperature and desiccation stress are associated with elevational range limits, incorporating data on microclimate conditions and phylogenetic relationships, and testing for intraspecific differences in individuals from different elevations on Mt. Rainier and from other mountains in Washington and Oregon.

## Methods

### Study area and species collection

Mt. Rainier (4392 m) lies ~180 km east of the Pacific Ocean in the northwest USA, has been protected as a National Park since 1899, and supports a succession of biomes from low-elevation deciduous forest, through to glaciers and permanent snow. The maximum diversity of *Nebria* occurs at 1300 m to 1400 m, where the elevational ranges of nine species overlap (Fig 1); however, only three to five species co-occur at any site ([21] Table 1). High-elevation *Nebria* are associated with permanent and temporary snowfields, while lower-elevation species are riparian. During the day, adults shelter beneath rocks on the edge of snowfields or streams, where multiple species are often found together. At night, beetles forage on the adjacent snowfields or stream edges; it is during this period that beetles experience the greatest microclimate variation–both within sites and across elevations. The life-cycles of most species are seasonal, with peak adult activity between May and September [21]. Exceptions are found in the two lowest-elevation species, *N*. *mannerheimi* (adults active from March to October) and *N*. *eschscholtzii* (adults active year-round) [21]. Larvae and overwintering adults persist in substrate beneath rocks and snow, in cold (around 0°C [25]), but thermally stable and humid microhabitats. Soil temperature variation in winter is dampened by the insulating effect of snow [26], as shown by records rarely exceeding a minimum of -3.8°C on Mt. Rainier [27]. Due to the thermal stability of winter microhabitats, and the observations that adult insects usually have poor thermal tolerance compared to juvenile life stages [28], and are likely exposed to both temperature and desiccation stress when they are surface active, we consider the adult stage the most relevant for testing elevational differences in acute physiological tolerances.

**Table 1:** Summary statistics for night-time temperature and relative humidity.

	Site 1	Site 2	Site 3	Site 4	Paradise (2014)	Paradise (10-yr)
Elevation (m a.s.l)	2180	1930	1610	970	1675	1675
Mean temperature (°C ± s.d)	4.3 ± 3.0	4.7 ± 2.7	5.6 ± 1.9	11.1 ± 2.7	11.4 ± 5.2	9.3 (6.3–11.4)
Absolute minimum (°C)	-1.9	-0.9	1.6	3.5	0.6	0.8 (-1.1–3.3)
Absolute maximum (°C)	12.3	10.6	13.2	21.7	20.6	19.1 (14.4–23.9)
Mean nightly minimum (°C)	3.3 ± 2.7	3.5 ± 2.4	5.6 ± 2.4	10.6 ± 2.9	9.5 ± 4.8	7.8 (4.9–9.5)
Mean nightly maximum (°C)	5.4 ± 3.3	6.1 ± 3.0	7.4 ± 2.5	15.0 ± 4.3	12.9 ± 4.8	11.1 (8.2–12.9)
Mean nightly fluctuation (°C)	2.1 ± 1	2.6 ± 1.1	1.8 ± 1.0	4.3 ± 1.8	3.5 ± 2.3	3.3 (2.7–3.9)
Mean RH (%)	98.2 ± 4.6	-	90.3 ± 9.5	89.0 ± 10.5	70.2 ± 25.2	79.7 (70.2–87.6)
Species present	***N*. *paradisi***	***N*. *paradisi***	***N*. *meanyi***	***N*. *sahlbergii***		
	***N*. *vandykei***	***N*. *vandykei***	***N*. *kincaidi***	***N*. *acuta***		
	*N*. *meanyi*	*N*. *kincaidi*	***N*. *gebleri***	***N*. *piperi***		
		*N*. *acuta*	*N*. *vandykei*	***N*. *mannerheimi***		
			*N*. *acuta*		*** ***	*** ***

Summary data are shown for the four primary collecting sites on Mt. Rainier from June 20^th^ to August 2^nd^, 2014 (44 nights). Night was defined, for each day, as between the hours of sunset and sunrise. Nightly fluctuations are calculated as the difference between the maximum and minimum temperature on a given night. Species present at each site are listed, and those for which thermal tolerance and desiccation resistance were measured are in bold. *Nebria crassicornis* was collected from 1910 m, *N*. *metallica* from 805 m (where it co-occurs with *N*. *piperi*, *N*. *mannerheimi*, and *N*. *acuta*), and *N*. *eschscholtzii* from 610 m (where it co-occurs with *N*. *mannerheimi*). Data are also shown for Paradise, Mt. Rainier (weather station PVC55, Northwest Avalanche Centre), for the same period in 2014 and for a 10-year (2005–2014) period. Data from 2014 are shown as mean ± s.d and 10-year data are shown as mean (range).

**Fig 2:**
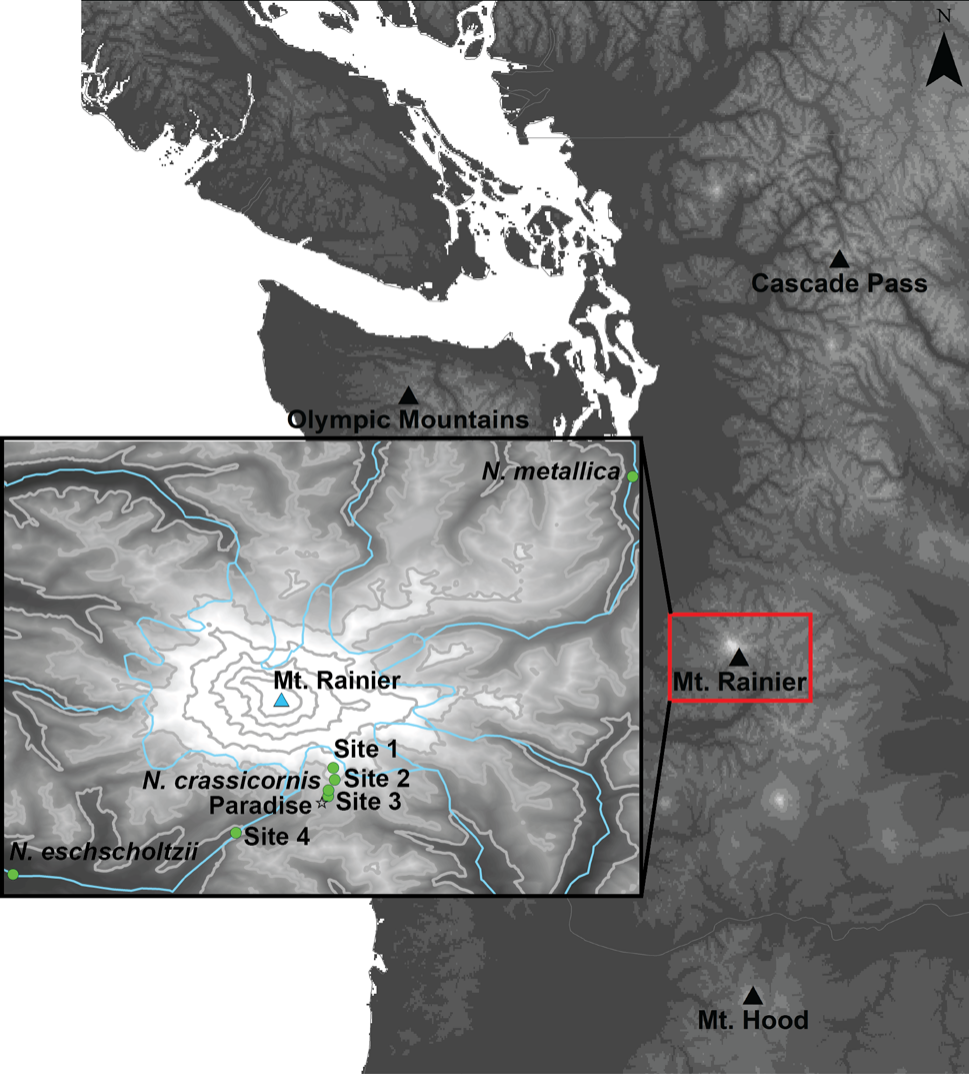
Map of the study area and collection sites. On Mt. Rainier, collection sites are indicated by circles; sites labelled with a species name are locations at which only one species was collected; details of the species collected at other sites are provided in Table 2. Sites 1–4 had iButtons deployed during the study period. The summit and the Paradise weather station are indicated with a triangle and star, respectively, and major rivers are shown with blue lines. Elevation in meters above sea level (m a.s.l.) is indicated in grey scale, and with 500 m contour lines.

Activity of all species is concentrated in the first three hours of darkness [29]. We collected adult beetles at night, by hand, during June and July, 2014. Nine species were collected from one of four sites (Table 1). Site 1 (2180 m) and 2 (1930 m) are snowfields overlying talus; Site 3 (1610 m) was a snow-covered stream, while Site 4 (970) was a snow-free stream edge. Multiple species were present at all sites, and species were collected at the site where they were most abundant. The remaining three species were not present at any of the primary collection sites and were collected from different snowfield (*N*. *crassicornis*, 1910 m) or stream-edge sites (*N*. *metallica*, 805 m; *N*. *eschscholtzii*, 610 m) (Fig 2).

Trait variation among species is a product of both macro- and micro-evolutionary processes and intraspecific variation can therefore affect apparent differences between species [30, 31]. We examined intraspecific variation in a subset of *Nebria* species. First, *N*. *paradisi* and *N*. *vandykei* were sampled from two sites on Mt. Rainier where they were abundant (Table 1). Second, *N*. *gebleri*, *N*. *kincaidi*, *N*. *meanyi*, *N*. *paradisi*, and *N*. *vandykei* were each sampled from mountain areas to the north (Cascade Pass or the Olympic Mountains) and south (Mt. Hood) of Mt. Rainier (Fig 2; Table 2). Ten individuals of each species were tested from each mountain, with the exception of *N*. *gebleri* and *N*. *kincaidi* from Mt Hood (5 and 7 individuals, respectively). An overview of our experiment can be found in S1 Appendix.

**Table 2:** Population details for intraspecific comparisons.

	South	Central	North	
	Mt. Hood	Mt, Rainier	Cascade Pass	Olympic Mountains
*N*. *vandykei*	2025 (10)	2180 (10)	1604 (10)	
		1930 (10)		
*N*. *paradisi*	2025 (10)	2180 (10)	1604 (10)	
		1930 (10)		
*N*. *meanyi*	1929 (10)	1610 (10)	1604 (10)	
*N*. *kincaidi*	1776 (7)	1610 (10)		1360 (10)
*N*. *gebleri*	1776 (5)	1610 (10)	180 (10)	

The elevations (m a.s.l) and sample sizes (in parentheses) are shown for species/populations used in intraspecific comparisons.

After collection, all beetles were kept in cool conditions (< 10°C) for one to three weeks before being transferred to 4°C for at least five days before experiments began. All the species in our study can be kept at this temperature for long periods of time with low mortality; it was thus deemed a suitable, low-stress temperature. Mortality is noticeably higher at warmer temperatures (e.g. 10°C).

Beetles were kept in single-species groups of 3 to 6 individuals (depending on species size) in 60 mL plastic cups with moistened cotton, and were fed weekly with diced mealworms (*Tenebrio molitor*). Samples from Mt. Rainier, the Olympic Mountains and Cascade Pass were collected under permits from the National Parks Service (USA) (permit numbers: MORA-2014-SCI-0006, OLYM-2015-SCI-0048 and NCCO-2015-SCI-0012, respectively). Permits were not required for collection at Mt. Hood.

### Elevational range

The elevational ranges for *Nebria* on Mt. Rainier were described by Kavanaugh [21]. To confirm that these ranges have not shifted substantially [32], and that the zonation pattern (i.e. the order of species along the elevational gradient) was the same, in June 2014 we carried out 29 transect surveys (100 m long, 5 m wide) between 960 m and 2650 m a.s.l. Transects ran parallel with the slope and were focused around the four primary collection sites (Fig 2, Table 1). Three to five transects were carried out near each of these sites, in addition to 10 transects above 2300 m, one at 2000 m (between Sites 1 and 2) and one at 1400 m (between Sites 3 and 4). No species were detected outside the ranges described by Kavanaugh [21], whose data were therefore used to rank species according to their relative upper or lower distribution limits (imperfectly correlated; Fig 1).

### Microclimate

Data loggers (iButtons: DS1922L for temperature and DS1923 for relative humidity; Maxim Integrated Products) were deployed at snowfield or stream edges at the four collection sites, in locations where beetles were commonly found (i.e. 2 loggers per site, see Table A in S2 Appendix). We recorded microclimate temperature (± 0.0625°C) every 30 minutes from June 3^rd^ to September 14^th^ 2014 (104 nights) and relative humidity (± 0.04%) every 30 minutes from June 20^th^ to August 2^nd^ (44 nights). Night-time (interval between sunset and sunrise) temperatures were extracted and used for data analysis as these are the most relevant for *Nebria* activity thresholds. The temperature logger from Site 3 was lost during the sampling period. For the purpose of comparison, the data presented correspond to the period in which all loggers were recording (total 44 nights). Summary statistics based on the full data are provided in Tables A and B in S2 Appendix. In addition to these microclimate observations, we obtained hourly temperature and relative humidity data over the study period for the Northwest Avalanche Centre weather station at Paradise (station number: PVC55), which is the closest weather station to collection Sites 1–3 [33]. We use these data to examine whether microhabitat conditions differ substantially from ambient and whether conditions recorded in 2014 reflect the normal range of temperature and humidity at Mt. Rainier.

### Thermal tolerance

We used a temperature ramping method to measure both cold and heat tolerance [34]. All experiments were conducted using a temperature-regulated water bath (TX150 R2, Grant Instruments, UK), which circulated 50% propylene glycol around six 50 mL beakers. In each experimental run, one beetle was placed in each beaker. A k-type thermocouple (Jaycar Electronics) was placed in two of the beakers and the beaker temperature was recorded every 10 seconds with a thermocouple data logger (TC-08, Pico Technology, UK).

Cold tolerance was tested with a ramping protocol of 10 minutes at 4°C, followed by cooling at 0.2°C/min. The critical thermal minimum (CT_min_) was scored as a loss of reactivity to moderate stimulation (tipping the beetle onto its side) ("chill coma" [35]). Beetles were removed from the experiment upon reaching their CT_min_ and placed in individual, 60 mL plastic containers with moistened cotton and a piece of mealworm for food. Beetles were allowed to recover for one week at 4°C before being tested for heat tolerance.

The heat tolerance protocol started with 10 minutes at 4°C, followed by a fast ramp (0.5°C/min) to 20°C, then a slow ramp (0.2°C/min). Critical thermal maximum (CT_max_) was scored as a loss of righting ability and onset of twitching [36]. Beetles were removed from the experiment upon reaching CT_max_ and returned to their individual container at 4°C. After 24 hours, each beetle was weighed and survival (righting and voluntary walking) was scored. Those that did not survive (8 of 302) were deemed to have exceeded their CT_max_ and were excluded from subsequent analyses. For each species, 10 individuals were scored for both cold and heat tolerance.

### Desiccation resistance

For insects, water loss rate accounts for most of the variation in desiccation resistance [16]. After thermal tolerance trials, individuals were allowed to recover for at least one week at 4°C, then randomly assigned to a temperature treatment (5°C or 10°C) for measurements of water loss rates. Five individuals per species were used at each temperature. Each beetle was weighed to 0.01 mg on an electronic microbalance (Sartorius Research) and transferred to a 12 mL plastic tube. This tube was covered with fine cloth mesh to permit air flow but prevent the beetle escaping. This tube was then sealed inside a 50 mL tube containing 5 g Indicating Drierite (W.A. Hammond Drierite Co., USA), which reduced relative humidity to < 5%. Tubes were then placed in an incubator at the assigned temperature. Each beetle was weighed after 12 h and 24 h, and we calculated mass-specific water loss rates (mgH_2_Og^-1^h^-1^) for each period. The ability of the beetle to right itself when placed on its back was recorded after 24 h. Righting ability was scored as “normal” if a beetle immediately righted itself, “slow”if it stayed on its back for between 2 and 5 seconds and “very slow” if righting took more than 5 seconds. However, as only 15 beetles showed a loss of righting ability, these data were not formally analysed. One *N*. *gebleri* was excluded from the analysis as it died during the experiment and had a water loss rate twice as high as other individuals. All individuals were starved for 24 h prior to the experiment, as faecal water loss can constitute a large and unpredictable component of total water loss in beetles [37].

### Phylogenetic reconstruction

We constructed a molecular phylogeny based on 1488 bp of the *cytochrome oxidase subunit I* (*CO1*) gene (Genbank Accessions: KU641243-KU641255) so that trait variation among species could be tested in a phylogenetic framework. The *COI* locus was amplified following published methods [38] using two sets of primers, LCO1490 (5’ GGTCAACAAATCATAAAGATATTGG) and HCO2198 (5’ taaacttcagggtgaccaaaaaatca) [39], and Jerry (5’ CAACATTTATTTTGATTTTTTGG) and Pat (5’ TCCAATGCACTAATCTGCCATATTA) [40]. PCR products were sequenced with each primer on a 3730 capillary sequencer using BigDye 3.1 chemistry (Applied Biosystems). These data were manually edited and aligned in Geneious v6.1.8 (Biomatters Ltd.), and mrmodeltest2 v2.3 [41] was used to estimate a substitution model based on the Akaike Information Criterion [42]. Model HKY + I + G was selected as the best model and used in separate data partitions representing each codon position. Beast v3.1.2 [43] was used to estimate a Bayesian phylogeny of the partitioned dataset based on four independent runs with the following conditions: 100 million steps with genealogies sampled every 10,000 steps under a strict molecular clock rate of 1.0. MCMC convergence was assessed using tracer v1.6 [44] and effective samples sizes for each parameter were confirmed to have values greater than 200. A 10% burn-in period was selected before calculating the maximum clade credibility tree in treeannotator v2.1.2 [43] (see Fig A in S3 Appendix).

### Statistical analysis

All analyses were carried out using in R 3.2.0 [45]. Using the microclimate data, we calculated mean nightly minimum and maximum temperatures, and nightly fluctuations (the difference between the minimum and maximum temperature on any given night). Ambient temperature lapse rates (°C reduction in temperature for every kilometer increase in elevation) on Mt. Rainier have been estimated at 3.5°Ckm^-1^ and 5.1°Ckm^-1^ for minimum and maximum temperatures, respectively [46]. To compare these with our microclimate measures, we calculated minimum and maximum temperature lapse rate using linear regressions of temperature on elevation. We note, however, that these are based on only four data points and should thus be treated cautiously. We used linear regression to test the relationship between hourly microhabitat and weather station temperatures.

CT_min_ and water loss rate data were log-transformed prior to analysis to improve the fit to a normal distribution. We tested for an association between critical thermal limits and elevational distribution, using the high-elevation (cold) range edge for comparison with CT_min_ and the low-elevation (warm) edge for CT_max_. Desiccation resistance could be greater for species with a higher range limit if increasing atmospheric aridity towards higher elevations controls species distributions [4]; alternatively, as desiccation rate is often temperature-sensitive, warmer conditions at low elevations might promote greater desiccation resistance among low-elevation species [47]. We therefore tested for a relationship between desiccation resistance (water loss rate) and both range edges. We used linear and quadratic regressions of trait means on elevation for cold and heat tolerance, respectively, based on visual inspection of the data and likelihood-ratio comparison of model fit. For desiccation resistance, we used a general linear model with treatment temperature included as a factor and both range edge limits as predictor variables. Body mass was included as a covariate in all initial analyses but, as it had no significant effect, was not included in the final analyses.

We performed three common tests for phylogenetic signal (non-independence) in each physiological trait as well as in range edge elevations [48]. First, we calculated Abouheif’s *C*_*mean*_ [49] with 999 permutations in the adephylo 1.1–6 package [50]. We also calculated Pagel’s λ [51] as this metric is robust for small phylogenies [48]. The value of λ represents the phylogenetic signal in the dataset, with λ = 0 indicating independent trait evolution (no phylogenetic signal) and λ = 1 being consistent with a Brownian motion model of trait evolution [51, 52]. We used likelihood ratios to test the null hypothesis of no phylogenetic signal, with the phytools 0.4–56 package [53]. Third, we directly compared the fit of Brownian motion, Ornstein-Uhlenbeck [54] and “white noise” (phylogenetic independence) models of evolution for each physiological trait and range edge limits using the geiger 2.03 package [55]. We used the sample size-corrected Akaike information criterion (AIC_c_) to assess model fit [56]. As we detected weak but significant phylogenetic signal for heat tolerance using Abouheif’s *C*_*mean*_ and a strong signal of phylogenetic non-independence for the low-elevation range edge (Table A in S3 Appendix), we used a phylogenetic generalized least squares regression (PGLS), implemented in the caper 0.5.2 package [57] to test the relationship between these two traits [58]. PGLS was fitted using a covariance matrix based on our *COI* phylogeny (Fig A in S3 Appendix) and using the maximum likelihood method to find the branch length transformation (λ) optimising the fit of the model to the data [59]. A phylogeny is a hypothesis about a clade’s evolutionary history, and phylogenetic uncertainty can introduce errors in comparative analyses [60]. Several nodes in our *COI* phylogeny had low support values (Fig A in S3 Appendix), so we repeated the above analyses using a phylogeny constructed from morphological data [61] and branch lengths generated using the method of Grafen [62] in the ape 3.2 package [63] (Fig B in S3 Appendix). Results were qualitatively similar between the two analyses, so only those based on *COI* are reported below. Results from the morphological phylogeny are provided in Table A in S3 Appendix.

To directly compare thermal tolerance among species without confounding effects of native site conditions [30], we tested for interspecific variation within sites at which multiple species were collected (Table 1) using analysis of variance.

Lastly, we tested the extent of intraspecific variation using linear models with species and location nested within species as factors. Location was coded as either high/low elevation for *N*. *vandykei* and *N*. *paradisi* on Mt. Rainier, or south/central/north for among-mountain comparisons. For the both analyses, CT_max_ was transformed using the logarithm of (*K* + 1)–*x*) where *K* was the maximum value recorded [64], to correct a strong negative skew.

All data are presented as mean values ± s.d.

## Results

### Microclimate

**Fig 3:**
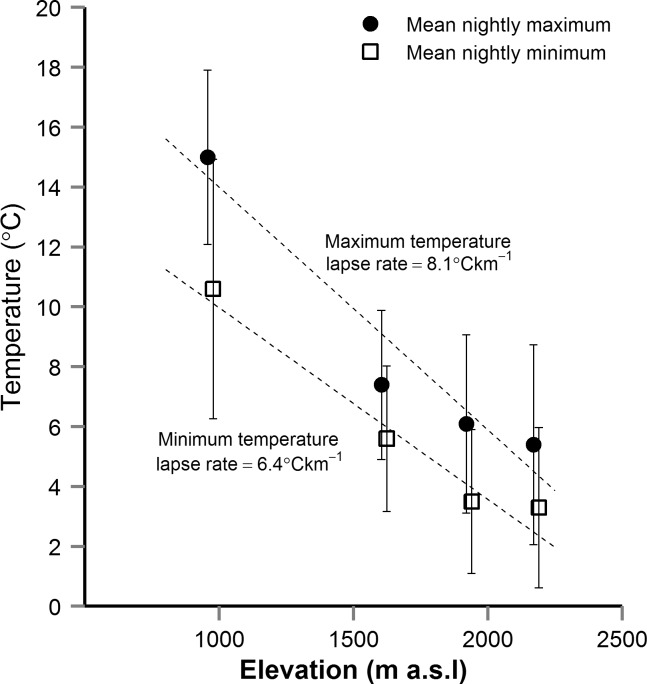
Microclimate temperature variation on Mt. Rainier. Mean (± s.d.) temperature variation among four *Nebria* collection sites on Mt. Rainier. Temperatures were recorded every 30 minutes for 44 days from June 20^th^ to August 2^nd^, 2014, with DS1922L iButtons. Mean nightly maximum (filled circles) and minimum (open squares) were calculated for each site using temperatures between the hours of sunset and sunrise.

Microclimate temperature data, from the iButtons, covered 44 days of the peak activity season of *Nebria* on Mt. Rainier. Across the four sites, temperature decreased approximately linearly with elevation, by 6.4°Ckm^-1^ (*r*^2^ = 0.96) and 8.1°Ckm^-1^ (*r*^2^ = 0.93) for mean nightly minimum and maximum, respectively (Fig 3). The nocturnal microclimate in snowfield- and stream-edge habitat is very stable, with a maximum nightly temperature fluctuation at a given site of 7.0°C and an average temperature fluctuation of 1.8°C (Site 3) to 4.3°C (Site 4) (Table 1). Further, across the whole study period, temperatures at a given site varied by no more than 18.2°C (Table 1). Even when recordings collected in September were included, the maximum temperature range was only 22°C (Table A in S2 Appendix). Relative humidity was generally high (mean 92.5% across all sites), and reached a minimum of 57% at Site 4, 61% at Site 2, and 75% at Site 1 (Table 1). At these three sites, humidity thus tended to increase with elevation and microhabitats had higher average relative humidity than ambient (weather station) conditions (Table 1).

Microhabitat temperatures were moderately to strongly correlated with ambient temperatures measured at the Paradise weather station (adjusted *R*^2^: Site 1: 0.84 (*n* = 608); Site 2: 0.83 (*n* = 608); Site 3: 0.82 (*n* = 290); Site 4: 0.63 (*n* = 610), for Sites 1–4, respectively). In all cases, the intercept of the regression line was above 0 and the slope less than 1, indicating reduced temperature variation in *Nebria* microhabitats; on cold nights, *Nebria* microhabitats tend to be warmer than ambient temperatures at Paradise (1676 m), while on “warm” nights (e.g. > 5°C for Sites 1–3, > 16°C for Site 4) they tend to be cooler. For June-July, 2014 was warmer and drier than 2005–2013 (Table 1); however, over the whole period of peak *Nebria* activity (June-September), conditions in 2014 fell within the 10-year norms (2014 mean: 6.1°C, nightly minimum: 8.1°C, nightly maximum: 11.1°C; 10-year mean: 6.8°C (range: 3.7–9.6), nightly minimum: 7.1°C (6.2–8.1), nightly maximum: 10.3°C (9.1–11.6); Table B in S2 Appendix).

### Elevational patterns of thermal tolerance and desiccation resistance

**Fig 4:**
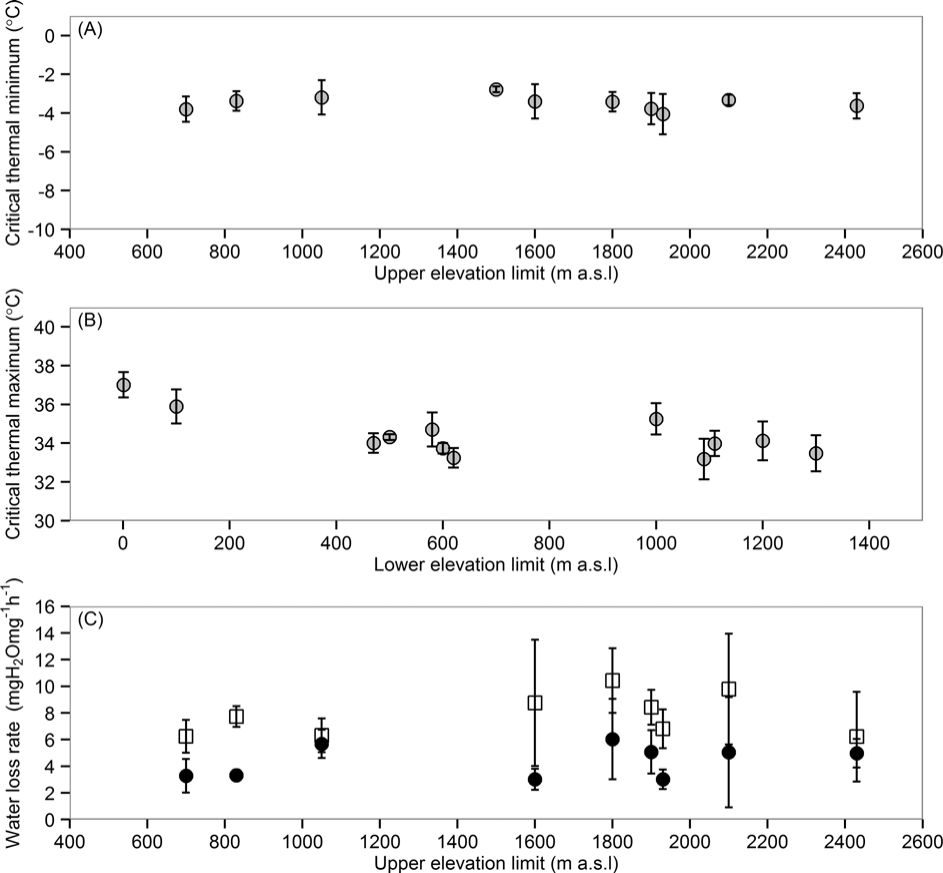
Associations between physiological traits and elevational range among 12 *Nebria* species from Mt. Rainier. (A) Cold tolerance and the high-elevation range edge (all *n* = 10); (B) heat tolerance and the low-elevation range edge (all *n* = 10); and (C) desiccation resistance and the high-elevation range edge (all *n* = 5; filled circles represent water loss rates at 5°C, while open squares are water loss rates at 10°C, measured over 24 h). In (B), three species have their lower elevation limit at 600 m, and two at 1100 m and have been repositioned for graphical purposes only; trait means for each species are also provided in Table C in S2 Appendix. Error bars are ± s.d.

Trait means for all physiological measures are provided in S2 Appendix. *Nebria* were able to maintain coordinated movements between -3.5 ± 0.8°C and 34.5 ± 1.5°C (*n* = 120). Cold tolerance varied little among species, ranging from -3.2 (*N*. *piperi*) to -4.1 (*N*. *kincaidi*), while heat tolerance ranged between 33.2 (*N*. *gebleri* and *N*. *kincaidi*) and 37.0°C (*N*. *eschscholtzii*) (Fig 4). For all species, these limits exceed both the minimum and maximum microclimate temperatures recorded over the study period (see Table 1 and Table C in S2 Appendix).

There was no association between cold tolerance and the upper elevation limit (*R*^2^ = 0.03, *p* = 0.589, *n* = 12; Fig 4A). Heat tolerance showed a significant curvilinear relationship to the lower elevation range edge (*R*^2^ = 0.67, *p =* 0.007, *n* = 12) (Fig 4B), reflecting a greater heat tolerance of *N*. *eschscholtzii* and *N*. *mannerheimi*, the two lowest-elevation species. Upper thermal limits were similar among the remaining species. Inclusion of phylogenetic information did not improve the model fit (λ optimised at 0) nor did the results change qualitatively under a Brownian motion model of trait evolution (λ set at 1) (*R*^2^ = 0.57, *p* = 0.023, df = 9).

Proportional water loss was faster in the first 12 h (7.8 ± 3.7% initial mass), compared to the second 12 h (6.9 ± 4.1% 12-h mass) (paired t-test: *t* = 9.14, df = 118, *p* < 0.001), but as the difference was small and similar among species, data were analysed from the whole 24-h period. Under desiccating conditions, beetles lost 5–40% of their body mass over 24 h and began to lose righting ability after losing 20% mass. Four beetles in the 5°C treatment and 11 beetles in the 10°C treatment showed some loss of righting ability after 24 h. These included four *N*. *gebleri* (21–34% mass loss), four *N*. *paradisi* (22–25% mass loss), four *N*. *acuta* (22–34% mass loss), and one each of *N*. *kincaidi* (21% mass loss), *N*. *vandykei* (27% mass loss), and *N*. *mannerheimi* (37% mass loss). Beetles lost water approximately 1.6 times faster at 10°C, compared to 5°C, translating to a mean difference of 0.17 mgH_2_Og^-1^h^-1^ and 7.5% difference in the proportion of body mass lost after 24 h (*F*_1,20_ = 32.84, *p* < 0.001). There was no association between the rate of water loss and elevational distribution, measured as either the upper (*F*_1,20_ = 3.05, *p* = 0.096) or lower (*F*_*1*,*20*_ *=* 0.15, *p* = 0.708) range edge (Fig 4C). We detected no evidence for phylogenetic signal in desiccation resistance (Table A in S3 Appendix).

### Interspecific variation in thermal tolerance at sympatric sites

When we compared species collected within a single site, cold tolerance was highly conserved among species (Site 1: *F*_1,18_ = 1.28, *p* = 0.273; Site 2: *F*_1,18_ *=* 1.23, *p* = 0.282; Site 3: *F*_2,27_ *=* 1.09, *p* = 0.351; Site 4: *F*_3,35_ *=* 1.81, *p* = 0.164; see Table D in S2 Appendix for pairwise comparisons). There was, however, significant interspecific variation in heat tolerance at the lower-elevation sites: at Site 3 (*F*_2,27_ *=* 10.85, *p* < 0.001) *N*. *meanyi* (35.3 ± 0.9°C) had a higher CT_max_ than *N*. *kincaidi* (33.2 ± 1.0°C; *p* = 0.001) and *N*. *gebleri* (33.2 ± 1.4°C; *p* = 0.001). At Site 5 (*F*_3,35_ *=* 5.19, *p* = 0.005), interspecific variation was driven largely by *N*. *mannerheimi* (35.9 ± 1.1°C), which had a significantly higher CT_max_ than both *N*. *acuta* (33.7 ± 1.7°C; *p* = 0.003) and *N*. *sahlbergii* (34.3 ± 1.3°C; *p* = 0.034) in pairwise comparisons.

### Intraspecific variation in thermal tolerance within and among mountains

**Fig 5:**
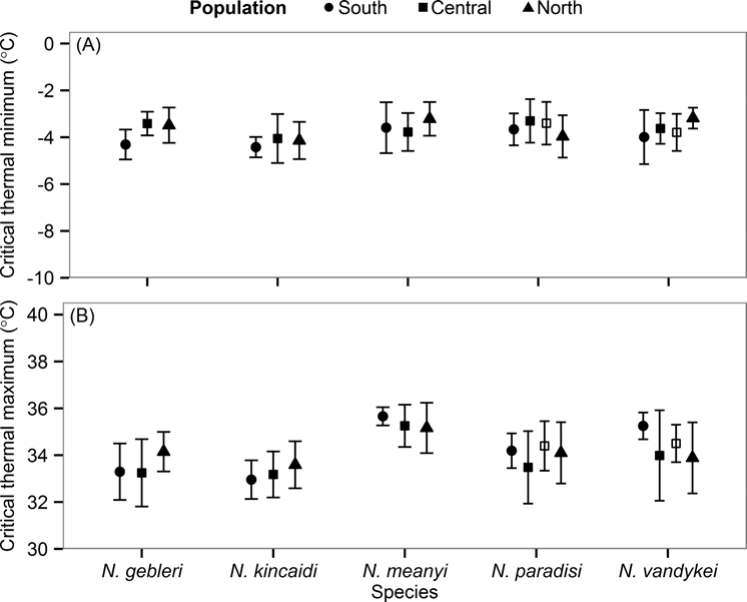
Intraspecific variation in thermal tolerance among five species of *Nebria*. (A) Cold tolerance and (B) heat tolerance. Different symbols correspond to population trait means (± s.d.) on different mountains (see Table 2 for details); for *N*. *paradisi* and *N*. *vandykei*, filled squares correspond to the low-elevation population on Mt. Rainier (Site 2) and open squares correspond to the high-elevation population on Mt. Rainier (Site 1).

For *N*. *vandykei* and *N*. *paradisi*, there was no difference in either cold or heat tolerance among high- and low-elevation populations on Mt. Rainier (CT_min_: *F*_1,36_ = 0.97, *p* = 0.390; CT_max_: *F*_1,36_ = 0.12, *p* = 0.889; Fig 5). Similarly, there was no significant variation in thermal tolerance (CT_min_: *F*_10,127_ = 1.62, *p* = 0.108; CT_max_: *F*_10,127_ = 1.59, *p* = 0.116) among populations from different mountains and, for both cold and heat tolerance, the majority of variation was partitioned among individuals (82% and 57% for CT_min_ and CT_max_, respectively; Fig 5).

## Discussion

Determining how the abiotic environment controls the ranges of species and whether this relates directly to measurable physiological variation is a critical issue for ecologists, with particular importance for predicting the outcomes of ongoing climatic change. In montane environments, where macroclimatic gradients are steep, physiological constraints are considered to be common determinants of elevational range limits [12, 13, 65]. In this study, we tested the hypothesis that physiological limits, measured as temperature and desiccation tolerance, were associated with the elevational ranges of the *Nebria* species on Mt. Rainier. Despite a steep decline in minimum microclimate temperatures with elevation, we found no evidence of variation in acute cold tolerance or desiccation resistance among species. Two low elevation species showed elevated heat tolerance, yet this provides limited evidence that heat tolerance governs the elevational turnover of *Nebria* species.

### Are thermal tolerance limits responsible for elevational turnover?

Recent syntheses of global variation in thermal tolerance traits highlight the close associations between cold tolerance and latitude among both vertebrate and invertebrate ectotherms [18, 20, 66]. Among the *Nebria* species tested here, we found remarkably little variation in cold tolerance in both inter- and intra-specific comparisons, which aligns with the overwhelming affinity for cold habitats within the *Nebria* radiation [21]. In comparison to cold tolerance, heat tolerance is typically more conserved among plant and animal lineages [67], shows less geographic variation ("Brett's rule" [68, 69]), and is thus less commonly invoked as a primary determinant of range limits [70]. Nevertheless, temperature (and associated physiological tolerance) has been identified as a dominant factor determining warm-edge range limits in a wide range of taxa [12, 71]. Among insects, critical thermal limits also coincide with maximum body temperatures predicted by biophysical models [72]. Heat tolerance variation among *Nebria* is negatively associated with elevation.

Two factors must be considered with respect to heat tolerance results. First, species traits are influenced by both the environmental conditions experienced across their range and their evolutionary history, with the latter favouring trait similarity among closely related species [1, 73–75]. By incorporating phylogenetic information, we took into account the non-independence of *Nebria* species [74] and found some evidence for phylogenetic signal in heat tolerance and the low-elevation range edge, suggesting that evolutionary constraints may play a role in structuring the Mt. Rainier *Nebria* assemblage. This observation was driven by *N*. *eschscholtzii* and *N*. *mannerheimi*, which are close relatives ([21] see Fig A in S3 Appendix). Although the association between heat tolerance and elevation remained significant after accounting for phylogenetic effects, the shared sub-montane distribution of these species (Fig 1) makes it difficult to disentangle the effects of common selection regimes from those of a shared evolutionary history in this dataset [76, 77]. A second factor to consider is that, among the remaining, strictly montane species, although we found significant interspecific variation in heat tolerance, this was not associated with a species’ elevational range. This information, coupled with the lack of variation in cold tolerance, strongly suggest that acute thermal limits in the adult life-stage are not responsible elevational range limits and, as a consequence, elevational species turnover in this system.

We must acknowledge several limitations of our experimental analysis of thermal tolerance limits, which were 1) the absence of thorough measures of intraspecific variation along elevational gradients, and 2) tests for the effect of laboratory acclimation. Populations spread across different mountains showed low variation in thermal limits relative to among-individual variation within populations, suggesting that thermal limits are conserved within species. However, thorough assessments of intraspecific variation along a single elevational gradient (particularly for those species whose range extends below the montane environment) would allow stronger conclusions to be made regarding the lability (or otherwise) of acute thermal tolerance traits within and among these species. Second, acclimation to laboratory conditions could differentially affect the thermal breadth of *Nebria* species in this study, as some studies have shown significant short-term thermal plasticity in insects [78]. However we consider laboratory acclimation to have minimal influence on our results, as short-term plasticity is typically induced by exposure to extreme temperatures (not benign temperature that are well within the natural range of the study species), and other studies of montane insects have found quite limited thermal plasticity (< 2°C) with respect to laboratory acclimation [79, 80].

### Desiccation resistance and microhabitat selection

All of the *Nebria* species included in our study rapidly desiccated under dry conditions, particularly at higher temperatures. Regulation of water balance is critical for maintaining physiological processes and there is considerable variation in desiccation resistance among insect species and populations [47, 81]. Several studies have found elevational variation in desiccation resistance, with some showing increasing desiccation resistance with elevation (e.g. for *Drosophila* [15])–a trend predicted by a general decrease in humidity with elevation [4]. However, there are, equally, several studies showing desiccation resistance decreases with elevation [82, 83]. Temperature and moisture have interacting effects on physiology [47]. For example, some species show temperature-dependent survival after desiccation stress [84] and temperature-dependent humidity preferences [85]. Desiccation rate is also strongly temperature-sensitive [47], complicating the expected relationship between elevation and desiccation resistance.

On Mt. Rainier, weather station records reveal a dry high-elevation environment: night-time atmospheric relative humidity for June and July, 2014, averaged 87% at 595 m a.s.l., 78% at 1680 m and just 41% at 3080 m [33]. These data contrast sharply with the high relative humidity we recorded near the substrate surface at stream and snowfield edges where beetles forage. Many high-elevation insects are closely associated with moist environments [4], and a shared behavioural preference for humid microhabitats that ameliorates the decrease in atmospheric moisture with elevation could explain the lack of interspecific variation in this trait.

### Life stage, chronic stress, and competition: missing pieces of the puzzle

With little variation in physiological traits, the question remains: what drives the marked differences in elevational distribution among *Nebria*? While elevational range margins can be a function of acute physiological thresholds [86], species turnover might also be driven by variation in other aspects of the physiological niche, species-specific habitat preferences and resources, or biotic interactions that constrain the distribution within the parameters of the physiological limits [8, 16, 87, 88].

In a laboratory study of larval development, Thiele [22] determined that a forest species, *Nebria brevicollis* requires cold temperatures (2–4°C) for larval development. How much this requirement varies among *Nebria* species is unknown, but studies in other insects have shown that elevational and latitudinal distributions can be limited by available heat budgets acting on larval development [89, 90]. Similarly, chronic exposure to moderately stressful conditions, under which individuals can maintain activity but accumulate sub-lethal injuries, can affect fitness through downstream effects on longevity and reproduction [91, 92]. Geographic variation in chronic temperature stress has rarely been examined in the context of species range limits, but offers a clear alternative pathway by which physiology might constrain distributions [93].

Habitat preferences could be important factors influencing the distribution of *Nebria* on Mt. Rainier, and indeed it is clear that some species occur exclusively in riparian or non-riparian habitats [94]. In a study of European *Nebria*, environmental conditions appeared to drive local distributional patterns [95]. However, it was unclear what factors could be directly responsible for microhabitat subdivision within habitat types (riparian or non-riparian sites). Quantifying differences in microhabitat and its association with abundance of species will be an important future direction in assessing range limits of *Nebria* on Mt. Rainier. It seems less likely that resource availability would play a significant role, as dietary analyses tend to suggest substantial overlap in scavenging carabid beetles [96], including *Nebria* in other montane regions [97].

Finally, for many species, climatic conditions and physiological constraints alone are unable to explain distribution limits, with biotic interactions constraining species to a subset of the environments which they could, physiologically, occupy [6, 8]. Spence [98] found no evidence for direct or indirect adult competition among two partially sympatric *Nebria* (e.g. both species are generalist scavengers, have the same activity patterns, and share diurnal refuges); Mann *et al*. [29] also found similar activity patterns among *N*. *vandykei*, *N*. *paradisi* and *N*. *crassicornis* on Mt. Rainier. However, Spence [98] suggests that larval competition might be important in structuring communities. When species differ in the conditions under which fitness is maximised, competitive exclusion can drive species turnover along environmental (e.g. elevational) gradients [8, 99]. If this is the case, the effect of competitive release should allow a species to expand its environmental (and physical) range when others species are absent [100]. On mountains to the south of Mt. Rainier, species diversity of *Nebria* declines. It is unclear at present whether range size expands in these less diverse communities. At a broad scale, species persist in the same macroclimatic zone across their geographic range, regardless of the assemblage [21], but more fine-scale study is required before the effects of competition can be properly assessed.

### Conclusions

Physiological traits, in particular thermal tolerance, are frequently inferred as proximal drivers of elevational range edges for insects and other ectotherms. This association has been used to predict and explain recent upslope shifts driven by climate warming and habitat change [32, 101, 102], contemporary range limits and patterns of species turnover along environmental gradients. We found no evidence for interspecific variation in either acute cold tolerance or desiccation resistance, and limited evidence that heat tolerance contributes to the elevational turnover of species. The elevational ranges of species in this system, and thus species turnover with elevation, is clearly driven by additional, unmeasured factors which might include physiological constraints such as temperature requirements for development or effects of chronic thermal stress, or biotic interactions.

## Supporting Information

S1 Appendix Experimental design flow chart. (DOCX)

S2 Appendix Summary statistics and pairwise comparisons. (DOCX)

S3 Appendix Phylogenetic trees and tests for phylogenetic signal. (DOCX)
