# Supplementary material for: Physiological Limits along an Elevational Gradient in a Radiation of Montane Ground Beetles
Source: PLoS One. 2016 Apr 4;11(4):e0151959. doi: 10.1371/journal.pone.0151959 (PMC4820226; doi:10.1371/journal.pone.0151959)
Supplement: S1 Appendix — (DOCX) [file pone.0151959.s001.docx]

**S1 Appendix. Experimental Design Flow Chart**


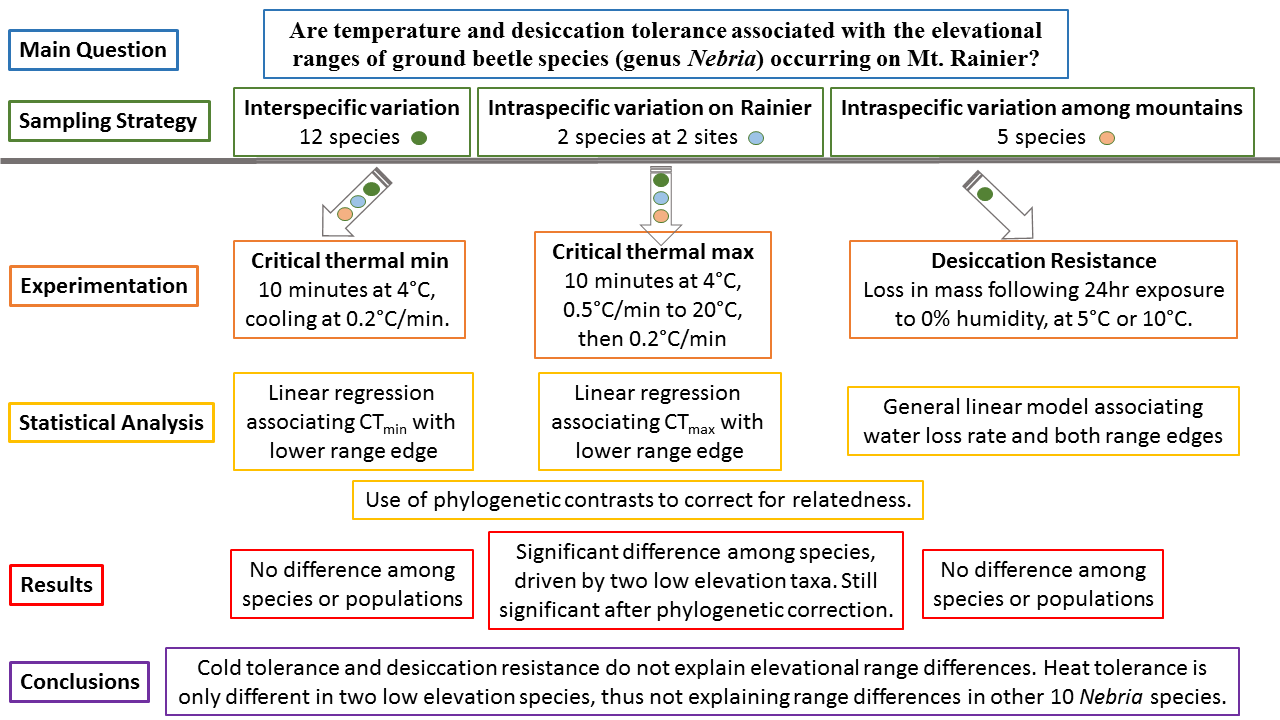


**Figure A.** Overview of research design employed to test whether physiological constraints determine the geographic ranges of ground beetle species on Mt. Rainier.
