# Supplementary material for: Physiological Limits along an Elevational Gradient in a Radiation of Montane Ground Beetles
Source: PLoS One. 2016 Apr 4;11(4):e0151959. doi: 10.1371/journal.pone.0151959 (PMC4820226; doi:10.1371/journal.pone.0151959)
Supplement: S2 Appendix — (DOCX) [file pone.0151959.s002.docx]

**S2 Appendix. Summary statistics and pairwise comparisons**

**Table A.** Summary statistics for night-time temperature and relative humidity at the four primary collecting sites on Mt. Rainier during June – September 2014. Logger A at each site was the primary data logger (iButton DS1922L), placed in a microhabitat location where beetles are commonly found. Logger B was a secondary logger, placed approximately 1 m away from Logger A, in an exposed microhabitat. Logger C at Sites 1 and 4 was an additional thermometer/hygrometer (iButton DS1923) placed in a similar microhabitat to Logger A. Species present at each site are listed, and those for which thermal tolerance and desiccation resistance were measured are in bold. *Nebria crassicornis* was collected from 1911 m, *N. metallica* from 804 m (where it co-occurs with *N. piperi*, *N. mannerheimi,* and *N. acuta*), and *N. eschscholtzii* from 610 m (co-occurring with *N. mannerheimi*).

|  | **Site 1** | | | **Site 2** | | **Site 3** | | **Site 4** | | |
| --- | --- | --- | --- | --- | --- | --- | --- | --- | --- | --- |
| Elevation (m a.s.l) | 2179 | | | 1929 | | 1613 | | 967 | | |
| Logger | **A** | **B** | **C** | **A** | **B** | **A** | **B** | **A** | **B** | **C** |
| Mean temperature (°C ± s.d) | 5.3 ± 2.9 | 7.6 ± 4.2 | 3.4 ± 1.6 | 6.5 ± 2.8 | 8.5 ± 3.8 | 6.5 ± 2.5 | 7.0 ± 3.5 | 13.6 ± 3.4 | 9.6 ± 2.7 | 12.0 ± 3.3 |
| Absolute minimum (°C) | -1.9 | -0.9 | -0.6 | -0.9 | -2.0 | 1.6 | 1.0 | 3.5 | 4.1 | 1.1 |
| Absolute maximum (°C) | 14.0 | 18.2 | 8.0 | 14.5 | 17.9 | 13.2 | 13.6 | 25.1 | 16.7 | 22.2 |
| Mean nightly minimum (°C ± s.d) | 4.0 ± 2.5 | 6.1 ± 3.9 | 1.9 ± 1.0 | 5.1 ± 2.5 | 7.2 ± 3.7 | 5.5 ± 2.5 | 5.5 ± 2.4 | 11.4 ± 2.6 | 7.9 ± 1.9 | 9.7 ± 2.2 |
| Mean nightly maximum (°C ± s.d) | 6.7 ± 3.2 | 9.6 ± 4.6 | 5.1 ± 2.0 | 8.1 ± 3.3 | 10.5 ± 3.8 | 7.4 ± 2.5 | 8.9 ± 3.4 | 16.4 ± 4.0 | 11.8 ± 3.5 | 15.1 ± 4.1 |
| Mean nightly fluctuation (°C ± s.d) | 2.7 ± 1.2 | 3.5 ± 1.8 | 3.2 ± 1.5 | 3.1 ± 1.3 | 3.3 ± 2.4 | 1.9 ± 1 | 3.5 ± 1.2 | 4.9 ± 2.1 | 3.9 ± 2.0 | 5.4 ± 3.1 |
| Number of nights recorded | 103 | 103 | 32 | 104 | 104 | 44 | 17 | 104 | 18 | 44 |
| Mean RH (% ± s.d) | 98.2 ± 4.6 | | | - | | 90.3 ± 9.5 | | 89.0 ± 10.6 | | |

**Table B.** Summary statistics for night-time temperature and relative humidity during June – September at Paradise (1675 m), Mt. Rainier (weather station PVC55, Northwest Avalanche Centre). Data shown are for 2014, and for a 10-year period (2005-2014), based on night-time observations of temperature and relative humidity. 2014 data are shown as mean ± sd and 10-year data are shown as mean (range)

|  | **2014** | **10-year** |
| --- | --- | --- |
| Mean temperature (°C) | 6.1 ± 6.5 | 6.8 (3.7 – 9.6) |
| Absolute minimum (°C) | -8.3 | -5.3 (-10.0 – 0.0) |
| Absolute maximum (°C) | 21.7 | 21.1 (17.8 – 23.9) |
| Mean nightly minimum (°C) | 8.1 ± 4.6 | 7.1 (6.2 – 8.1) |
| Mean nightly maximum (°C) | 11.1 ± 4.9 | 10.3 (9.1 – 11.6) |
| Mean nightly fluctuation (°C) | 3.1 ± 2.1 | 3.3 (2.6 – 3.9) |
| Mean RH (%) | 80.5 ± 22.9 | 80.9 (75.4 – 86.1) |

**Table C.** Trait means (± s.d.) for thermal tolerance and desiccation resistance for each *Nebria* species included in the study; population elevation is the elevation of the population for which traits were measured. Mass-specific water loss rates (mgH_2_Og^-1^h^-1^) and total desiccation (% initial mass, in brackets) at 5°C and 10°C at < 5% relative humidity, calculated after 24 hours. All *n* = 10 for CT_min_ and CT_max_, and *n* = 5 for all water loss data.

|  |  |  |  | **Water loss rates  [mgH_2_Og^-1^h^-1^ (% initial mass loss)]** | |
| --- | --- | --- | --- | --- | --- |
| **Species** | **Population** | **CT_min_ (°C)** | **CT_max_ (°C)** | **5°C** | **10°C** |
| *N. eschscholtzii* | Mt. Rainier | -3.8 ± 0.7 | 37.0 ± 1.2 | 3.3 ± 1.3  (7.9 ± 3.0) | 6.2 ± 1.2  (14.0 ± 3.0) |
| *N. metallica* | Mt. Rainier | -3.4 ± 0.5 | 34.0 ± 0.8 | 3.3 ± 0.4  (8.0 ± 1.0) | 7.7 ± 0.8  (18.6 ± 1.9) |
| *N. piperi* | Mt. Rainier | -3.2 ± 0.9 | 34.7 ± 0.8 | 5.7 ± 1.1  (13.6 ± 2.6) | 6.3 ± 1.3  (15.2 ± 3.1) |
| *N. mannerheimi* | Mt. Rainier | -3.4 ± 0.9 | 35.9 ± 1.1 | 3.0 ± 0.8  (7.3 ± 1.9) | 8.8 ± 4.7  (21.0 ± 11.4) |
| *N. sahlbergii* | Mt. Rainier | -2.8 ± 0.1 | 34.3 ± 1.3 | 5.7 ± 2.7  (13.8 ± 6.6) |  |
| *N. acuta* | Mt. Rainier | -3.3 ± 0.3 | 33.7 ± 1.7 | 5.1 ± 4.1  (12.1 ± 9.9) | 9.8 ± 4.2  (23.5 ± 10.0) |
| *N. gebleri* | Mt. Rainier | -3.4 ± 0.5 | 33.2 ± 1.4 | 6.0 ± 3.0  (14.5 ± 7.3) | 10.4 ± 2.4  (25.0 ± 5.8) |
|  | Mt. Hood | -4.3 ± 0.6 | 33.3 ± 1.2 |  |  |
|  | Cascade Pass | -3.5 ± 0.8 | 34.1 ± 0.8 |  |  |
| *N. meanyi* | Mt. Rainier | -3.8 ± 0.8 | 35.3 ± 0.9 | 5.1 ± 1.6  (12.2 ± 3.9) | 8.4 ± 1.3  (20.2 ± 3.1) |
|  | Mt. Hood | -3.6 ± 1.1 | 35.7 ± 0.4 |  |  |
|  | Cascade Pass | -3.2 ± 0.7 | 35.2 ± 1.1 |  |  |
| *N. kincaidi* | Mt. Rainier | -4.1 ± 1.0 | 33.2 ± 1.0 | 3.0 ± 0.7  (7.2 ± 1.8) | 6.8 ± 1.5  (16.4 ± 3.5) |
|  | Mt. Hood | -4.4 ± 0.4 | 33.0 ± 0.8 |  |  |
|  | Olympic Mountains | -4.1 ± 0.8 | 33.6 ± 1.0 |  |  |
| *N. crassicornis* | Mt. Rainier | -3.9 ± 1.0 | 34.1 ± 1.3 | 4.1 ± 0.8  (9.8 ± 1.9) | 5.5 ± 1.7  (13.3 ± 4.1) |
| *N. vandykei* | Mt. Rainier (low) | -3.6 ± 0.7 | 34.0 ± 1.9 | 5.0 ± 1.1  (11.9 ± 2.6) | 6.2 ± 3.4  (14.9 ± 8.1) |
|  | Mt. Rainier (high) | -3.8 ± 0.8 | 34.5 ± 0.8 |  |  |
|  | Mt. Hood | -4.0 ± 1.2 | 35.2 ± 0.6 |  |  |
|  | Cascade Pass | -4.0 ± 0.9 | 33.9 ± 1.5 |  |  |
| *N. paradisi* | Mt. Rainier (low) | -3.3 ± 0.9 | 33.5 ± 1.5 | 5.7 ± 2.8  (13.7 ± 6.7) | 8.6 ± 1.9  (20.6 ± 4.6) |
|  | Mt. Rainier (high) | -3.4 ± 0.9 | 34.4 ± 1.1 |  |  |
|  | Mt. Hood | -3.7 ± 0.7 | 34.2 ± 0.7 |  |  |
|  | Cascade Pass | -3.3 ± 0.9 | 34.1 ± 1.3 |  |  |

**Table D.** Pairwise comparisons of thermal tolerance limits at Sites 3 and 5. Mean differences (absolute values) among species for cold tolerance (log-transformed) are shown below the line, and for heat tolerance above the line. Significant differences (*p* < 0.05), after adjusting for multiple comparisons, are in bold.

*Site 3 Site 5*

| **CTmax**  **CTmin** | *N. kincaidi* | *N. meanyi* | *N. gebleri* |  | **CTmax**  **CTmin** | *N. mannerheimi* | *N. acuta* | *N. piperi* | *N. sahlbergi* |
| --- | --- | --- | --- | --- | --- | --- | --- | --- | --- |
| *N. kincaidi* |  | **2.08** | 0.07 |  | *N. mannerheimi* |  | **2.16** | 1.19 | **1.69** |
| *N. meanyi* | 0.03 |  | **2.01** |  | *N. acuta* | 0.00 |  | 0.97 | 0.47 |
| *N. gebleri* | 0.06 | 0.04 |  |  | *N. piperi* | 0.03 | 0.03 |  | 0.50 |
|  |  |  |  |  | *N. sahlbergii* | 0.08 | 0.08 | 0.05 |  |
