# Supplementary material for: Physiological Limits along an Elevational Gradient in a Radiation of Montane Ground Beetles
Source: PLoS One. 2016 Apr 4;11(4):e0151959. doi: 10.1371/journal.pone.0151959 (PMC4820226; doi:10.1371/journal.pone.0151959)
Supplement: S3 Appendix — (DOCX) [file pone.0151959.s003.docx]

**S3 Appendix. Phylogenetic trees and tests for phylogenetic signal**





**Fig A**. Bayesian maximum clade credibility tree for the 12 *Nebria* species on Mt. Rainier, based on 1,488 bp of the mitochondrial *CO1* gene. *Nippononebria campbelli* was included as an outgroup and the tree was generated in BEAST v3.1.2 using the HKY + I + G substitution model and a strict molecular clock, with data partitioned by codon position. One sequence per species was used to generate the gene tree. Nodal support values represent Bayesian posterior probabilities.

**

**

**Fig B.** Phylogeny for the *Nebria* on Mt Rainier, and *Nippononebria* *campbelli,* using morphological data, as proposed by Kavanaugh (1).

**Table A.** Tests for phylogenetic signal in physiological traits and range limits among *Nebria* from Mt. Rainier, based on both the *COI* and morphological phylogenies. Phylogenetic non-independence was tested with Abouheif’s *C_mean_*, Pagel’s λ and by comparison of three models of trait evolution: Brownian motion, Ornstein-Uhlenbeck and “white noise” (phylogenetically independent trait evolution). For model comparisons, the model with the lowest Akaike information criterion (AICc) for each trait is shown in bold.

| **Variable** | **Abouheif's *C_mean_*** | **Pagel's λ** | **Model comparison** | | |
| --- | --- | --- | --- | --- | --- |
|  |  |  | **Brownian motion** | **Ornstein–Uhlenbeck** | **White noise** |
| *Molecular phylogeny* |  |  |  |  |  |
| Log(CT_min_) | -0.08 | 6.69x10^-5^ | -16.09 | -21.37 | **-37.21** |
| CT_max_ | 0.33* | 0.89 | 42.82 | 42.89 | **41.59** |
| Log(mass-specific water loss rate, 5°C) | 0.10 | 0.28 | -12.16 | -10.81 | **-12.91** |
| Log(mass-specific water loss rate, 10°C) | 0.09 | 7.28x10^-5^ | -11.59 | -11.43 | **-17.07** |
| High-elevation range edge | 0.01 | 6.69x10^-5^ | **178.09** | 181.49 | 183.61 |
| Low-elevation range edge | 0.43** | 0.99** | 205.36 | 202.58 | **194.76** |
| *Morphological phylogeny* |  |  |  |  |  |
| Log(CT_min_) | -0.08 | 7.27x10^-5^ | -26.04 | -28.75 | -**37.21** |
| CT_max_ | 0.33 | 0.91 | **40.95** | 41.99 | 41.59 |
| Log(mass-specific water loss rate, 5°C) | 0.05 | 7.27x10^-5^ | -4.15 | -6.06 | **-12.91** |
| Log(mass-specific water loss rate, 10°C) | -0.06 | 7.27x10^-5^ | -7.06 | -9.12 | -**17.07** |
| High-elevation range edge | 0.16 | 0.24 | **183.22** | 184.28 | 183.61 |
| Low-elevation range edge | 0.41** | 0.69 | 201.58 | 200.15 | **194.76** |

**p*<0.05; ***p* <0.02

**References**

1. Kavanaugh DH. Investigations on present climatic refugia in North America through studies on the distributions of carabid beetles: concepts, methodology and prospectus. In: Erwin TL, Ball GE, Whitehead DR, Halpern AL, editors. Carabid Beetles. The Hague: Dr. W. Junk bv Publishers; 1979. p. 369-81.
